# Accurate sex prediction of cisgender and transgender individuals without brain size bias

**DOI:** 10.1038/s41598-023-37508-z

**Published:** 2023-08-24

**Authors:** Lisa Wiersch, Sami Hamdan, Felix Hoffstaedter, Mikhail Votinov, Ute Habel, Benjamin Clemens, Birgit Derntl, Simon B. Eickhoff, Kaustubh R. Patil, Susanne Weis

**Affiliations:** 1 Institute of Systems Neuroscience, Heinrich Heine University Düsseldorf, Düsseldorf, Germany; 2 Institute of Neuroscience and Medicine (INM-7: Brain and Behaviour), Research Centre Jülich, Jülich, Germany; 3 Department of Psychiatry, Psychotherapy and Psychosomatics, Faculty of Medicine, RWTH Aachen University, Aachen, Germany; 4 Institute of Neuroscience and Medicine (INM-10: Brain Structure-Function Relationships), Research Centre Jülich, Jülich, Germany; 5 Department of Psychiatry and Psychotherapy, Tübingen Center for Mental Health, University of Tübingen, Tübingen, Germany; 6 LEAD Graduate School and Research Network, University of Tübingen, Tübingen, Germany

**Keywords:** Neuroscience, Computational neuroscience

## Abstract

The increasing use of machine learning approaches on neuroimaging data comes with the important concern of confounding variables which might lead to biased predictions and in turn spurious conclusions about the relationship between the features and the target. A prominent example is the brain size difference between women and men. This difference in total intracranial volume (TIV) can cause bias when employing machine learning approaches for the investigation of sex differences in brain morphology. A TIV-biased model will not capture qualitative sex differences in brain organization but rather learn to classify an individual’s sex based on brain size differences, thus leading to spurious and misleading conclusions, for example when comparing brain morphology between cisgender- and transgender individuals. In this study, TIV bias in sex classification models applied to cis- and transgender individuals was systematically investigated by controlling for TIV either through featurewise confound removal or by matching the training samples for TIV. Our results provide strong evidence that models not biased by TIV can classify the sex of both cis- and transgender individuals with high accuracy, highlighting the importance of appropriate modeling to avoid bias in automated decision making.

## Introduction

Machine Learning (ML) approaches have become increasingly popular in medical imaging, especially for neuroimaging data^1–3^. Previous studies applying ML approaches to neuroimaging data coming from individuals with mental and neurodegenerative disorders have provided valuable insights into the complex mechanisms underlying psychopathology^4–6^. The ability of ML models to make predictions about previously unseen individual subjects has expanded the field from population-based analyses to investigation of individualized biomarkers^5,6^. However, it is important to ensure that predictions are not confounded by variables that are not part of the causal pathway of interest, but are associated with both the features the model was trained on and the target^6,7^, as results from confounded analyses might potentially lead to inaccurate and spurious conclusions^8,9^. Using brain size bias in sex classification as an example, the present study examines which confound removal strategy is most suitable to achieve high classification accuracy while effectively removing brain size bias^8–10^.

ML approaches have been successfully applied to the study of sex differences in the brain by training a classifier to predict sex based on features derived from structural brain imaging data, e.g. regional grey matter volume (GMV). Such a sex classifier is expected to capture multivariate brain organizational patterns that differ between the sexes. High classification accuracies on out-of-sample data^11,12^ are then taken as evidence for qualitative sex differences in the brain^13,14^. So far, studies using sex classification approaches based on structural brain imaging data achieved classification accuracies ranging from 82 up to 94%^11,12,15–17^. However, a sex classifier biased by brain size (measured as total intracranial volume, TIV^18,19^) will result in predictions that are driven by TIV differences rather than actual sex differences in brain structure^9,10,20^. As a result, a TIV-biased model will classify individuals with higher TIV as males and individuals with lower TIV as females, while making more mistakes for individuals with intermediate TIV.

The use of such a TIV-biased sex classifier is particularly problematic when analyzing data of individuals for whom local and global brain structural alterations have been reported, such as those with "gender incongruence," where a person's sex and gender identity differ^21^*.* In the present paper, following the linguistic guidelines provided by the Professional Association of Transgender Health^22^, the term “sex” is used to refer to the sex that a person was assigned at birth based on their anatomical sexual characteristics, whereas the term “gender (identity)” is used to denote the subjective identification of an individual as female, male, or one of the other gender identities which might be also fluid or non-binary. While the coherence of sex and gender is termed cisgender for cisgender men and women (CM, CW), gender incongruent individuals are denoted as transgender men and women (TM, TW,^21^).

To date, it is not yet fully understood if and to which extent local and global brain organization of transgender individuals is driven by factors matching their gender identity on top of those matching their sex. So far, studies contrasting groups of cisgender and transgender individuals reported regional GMV differences in the putamen^23^, insula^16^ as well as in surface areas, cortical and subcortical brain volumes^24^. Additionally, transgender individuals undergoing cross-sex hormone treatment (CHT) were reported to show structural alterations in the hypothalamus and the third ventricle^25^. Thus, there is some evidence indicating that transgender individuals display local brain volume differences^24,26–28^. Extending the results of group studies contrasting cisgender and transgender individuals, sex classification approaches—building a classifier on cisgender individuals’ data and then applying it to transgender individuals—have reported reduced sex classification accuracies for transgender compared to cisgender samples (76.2% vs. 82.6%^17^; 61.5% vs. 93.2–94.9%^16^). Higher rates of misclassification of sex in transgender as opposed to cisgender individuals have been taken to indicate that transgender brains might differ from those typical for their sex, implying an interaction between sex and gender at the neuroanatomical level^16,17,29^. However, before such conclusions can be drawn, biases that can influence a sex classifier must be taken into account, particularly those related to TIV^18,19^. It is crucial to be aware of the impact of local and global structural brain alterations that can lead to increases or decreases of TIV resulting in the TIV of transgender individuals falling between TIV of cisgender women and men^25^. Consequently, the predictions of a TIV-biased classifier might erroneously be interpreted as evidence for transgender brain organization to align with gender identity as has been reported before^16,29^.

**Figure 1:**
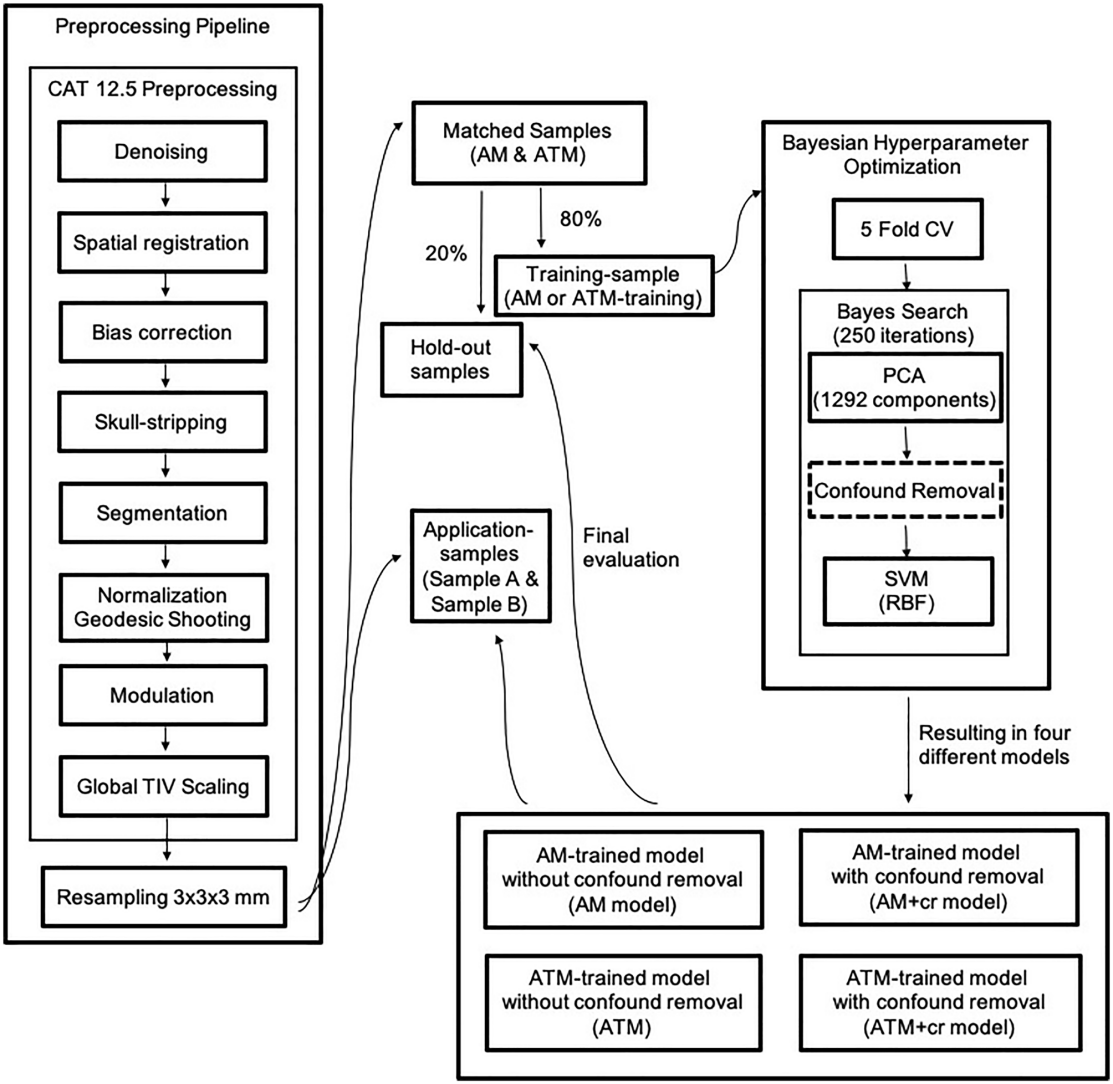
Analysis pipeline. Workflow of the sex classification analysis.

Here, we investigate the impact of TIV bias by examining two approaches to control for confounding effects of TIV^10^ in sex classification to evaluate which approach is most suited to account for TIV bias in the present sex classification analysis. We compare two statistically different approaches of controlling for TIV bias in comparison to a baseline model that does not account for the influence of TIV. For the first approach, we built debiased models through featurewise confound control by removing confounding effects of TIV during training (Fig. 1,^20,30^). In the second approach, we trained models on a stratified sample where women and men were matched for TIV. Model performance and TIV bias were assessed on hold-out samples of cisgender individuals to compare performance of the biased to the debiased models. We hypothesized that a TIV-biased model should achieve high performance but also exhibit a biased output pattern. In contrast, a model not biased by TIV will likely exhibit a drop in classification accuracy. However, importantly, misclassifications of such a model should be largely independent of TIV. In the final step, the debiased models were applied to application samples comprising both cisgender and transgender individuals to examine whether models without a TIV bias provide any evidence for an interaction of sex and gender influences on structural brain organization, as previously suggested^17^.

## Results

Classifiers employing Support Vector Machine (SVM) models with radial basis function kernel (rbf) were trained on whole-brain voxelwise GMV data of two large, non-overlapping cisgender samples to classify sex assigned at birth. In the first sample, women and men were matched for age (AM sample) to create a sample with a natural occurring TIV-distribution (Fig. S1 and Table S1). As a baseline, we trained the first model on this sample without any control for TIV bias (AM model), following the methodology of a previous study^16^. We then compared the baseline model to other models, which integrated two different approaches for confound control in order to assess which approach successfully removes TIV bias while accurately classifying sex. For the first approach, a ML model was also trained on the AM sample, but additionally controlled for TIV bias by featurewise confound removal (AM+cr model), while the third model comprised stratification for TIV by training the model on a sample of women and men who were matched for both age and TIV (ATM; see Fig. S1 and Table S1 for demographic details and TIV distribution of the samples). While the third model was trained on the ATM sample without additional TIV-control (ATM model) to evaluate stratification in itself, the fourth model employed a combination of both approaches to assess whether the addition of featurewise confound removal might further improve results (AM+cr model, Fig. 1). Subsequently, all models were calibrated to ensure that the prediction probabilities of the models match the respective class label (Figs. S2 and S3, Supplementary Results, https://scikit-learn.org/stable/modules/calibration.html#calibration). To evaluate model performance on hold-out data, each sample (AM and ATM) was split into a training sample (80%) and a hold-out sample (20%). As the two approaches—featurewise confound removal and stratification by matching—might exhibit differences in model performance since they are based on different statistical processes^8^, all four models were evaluated on both AM and ATM hold-out samples. This allowed for a thorough understanding of model behavior and evaluation of whether both approaches successfully remove TIV bias. Assessing model performance on the first sample (AM hold-out sample), which exhibits a naturally occurring TIV-distribution among women and men, enables a realistic evaluation of the model’s effectiveness in broader populations beyond those included in the present study. In turn, the ATM hold-out sample enables a more in-depth evaluation of the model performance, as it displays no significant difference in TIV between women and men. Consequently, an accurate model performance for the ATM hold-out sample indicates a non-TIV-biased model behavior as the model classifies a person’s sex based on other features than TIV, providing a “confound-free accuracy”^31^. Additionally, the models were tested on two independent application samples comprising transgender and cisgender individuals (sample A, sample B, see Fig. S1 and Table S1 for demographic details and TIV distribution of the samples).

### Evidence for TIV bias in the AM model

**Figure 2:**
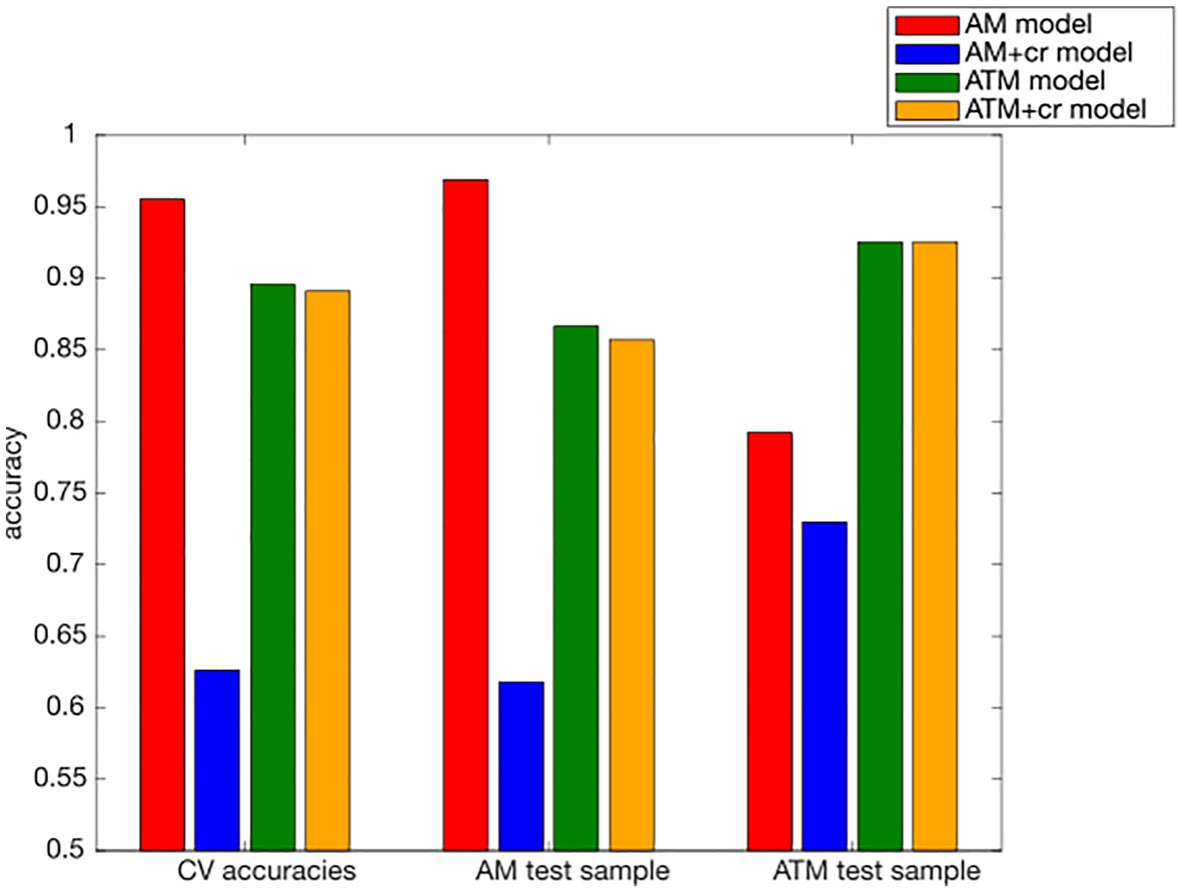
Sex classification accuracy. Accuracy values of the four different models for the cross validation (CV)-folds and applied to the AM and ATM hold-out sample.

**Figure 3:**
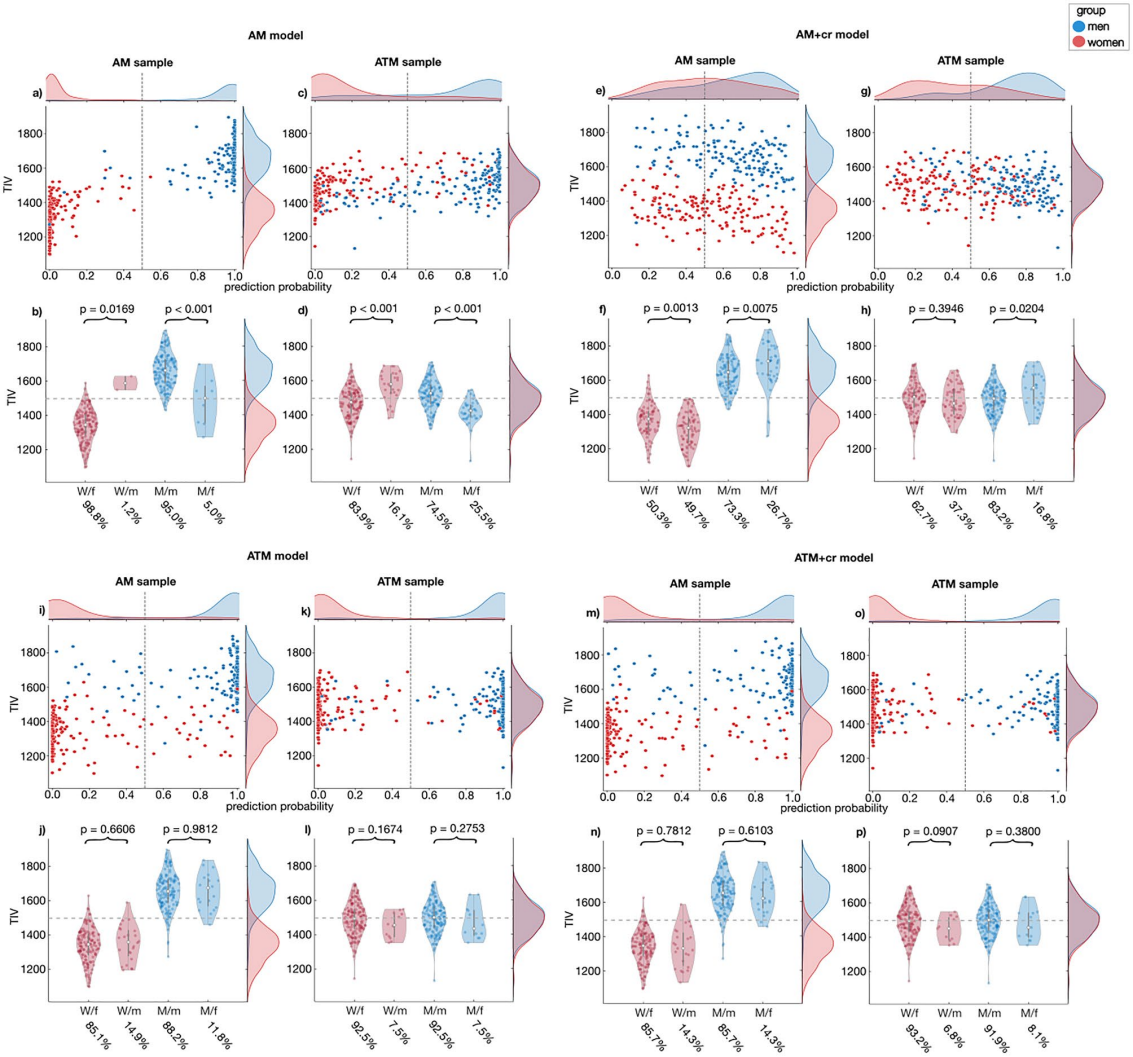
Association between prediction probability and TIV. Prediction probability (**a**, **c**, **e**, **g**, **i**, **k**, **m**, **o**) and TIV distribution (**b**, **d**, **f**, **h**, **j**, **l**, **n**, **p**) of sex congruently and incongruently classified women (red) and men (blue) of all four models applied to the AM and ATM hold-out sample. (W/f: women classified as female; W/m: women classified as male; M/m: men classified as male; M/f: men classified as female).

The application of the AM model to the AM hold-out sample resulted in a high classification accuracy of 96.89% (Table 1, Table S2, and Fig. 2). Accordingly, the assigned probability of being classified as male (prediction probability) was higher for men than for women (Fig. 3a). The comparison of TIV distributions revealed that men who were classified congruently with their sex as male had a significantly higher TIV than incongruently classified men (Fig. 3b). Similarly, women classified incongruently with their sex as male on average had a higher TIV than congruently classified women, even though this difference was not significant (details in Table 2).

**Table 1:** Model performance of all models applied to the hold-out and application samples (* Balanced Accuracy).

	AM model	AM+cr model	ATM model	AM+cr model
Model performance for the AM hold-out sample				
Recall:	0.9503	0.7329	0.8820	0.8571
Specificity:	0.9876	0.5031	0.8509	0.8571
F1:	0.9684	0.6574	0.8685	0.8571
BA*:	0.9689	0.6180	0.8665	0.8571
Model performance for the ATM hold-out sample				
Recall:	0.7453	0.8323	0.9255	0.9193
Specificity:	0.8385	0.6273	0.9255	0.9317
F1:	0.7818	0.7549	0.9255	0.9250
BA*:	0.7919	0.7298	0.9255	0.9255
Model performance for sample A				
Recall:	0.9474	0.7895	1	0.9474
Specificity:	0.8276	0.7241	0.8276	0.8448
F1:	0.8926	0.7627	0.9194	0.9000
BA*:	0.8875	0.7568	0.9138	0.8961
Model performance for sample B				
Recall:	0.8889	0.8333	0.9722	0.8889
Specificity:	0.9608	0.5882	0.9020	0.9020
F1:	0.9143	0.6897	0.9211	0.8767
BA*:	0.9248	0.7108	0.9371	0.8954

Model performance of all models applied to the hold-out and application samples.

**Table 2:** Wilcoxon rank sum tests of the hold-out samples.

	TIV women classified as female versus classified as male	TIV men classified as male versus classified as female
AM hold-out sample		
AM model	*T* = 12,722, z = − 2.3885, *p* = 0.0169, η^2^ = 0.0354	*T* = 12,829, z = 3.3879, *p* < 0.001, η^2^ = 0.0713
AM+cr model	*T* = 7514, z = 3.2204, *p* = 0.0013, η^2^ = 0.0644	*T* = 8858, z = − 2.6727, *p* = 0.0075, η^2^ = 0.0444
ATM model	*T* = 11,004, z = − 0.4390, *p* = 0.6606, η^2^ = 0.0012	*T* = 11,507, z = 0.0236, *p* = 0.9812, η^2^ < 0.001
AM+cr model	*T* = 11,236, z = 0.2778, *p* = 0.7812, η^2^ < 0.001	*T* = 11,284, z = 0.5097, *p* = 0.6103, η^2^ = 0.0016
ATM hold-out sample		
AM model	*T* = 9908, z = − 4.7156, *p* < 0.001, η^2^ = 0.1381	*T* = 11,325, z = 6.2257, *p* < 0.001, η^2^ = 0.2407
AM+cr model	*T* = 8425, z = 0.8513, p = 0.3946, η2 = 0.0045	*T* = 10,341, z = − 2.3190, *p* = 0.0204, η^2^ = 0.0334
ATM model	*T* = 12,284, z = 1.3806, *p* = 0.1674, η^2^ = 0.0118	*T* = 12,239, z = 1.0910, *p* = 0.2753, η^2^ = 0.0074
AM+cr model	*T* = 12,403, z = 1.6918, *p* = 0.0907, η^2^ = 0.0178	*T* = 12,130, z = 0.8780, *p* = 0.3800, η^2^ = 0.0048

Comparison of individuals classified as female versus male (Wilcoxon rank sum tests) for the AM and ATM sample.

When applied to the ATM hold-out sample, the AM model resulted in a much lower classification accuracy of 79.19% (Tables 1 and S2), presumably as the model could not rely on TIV for classifying in the ATM sample. Still, we observed a similar pattern as above, with men having a higher prediction probability than women (Fig. 3c), significantly higher TIV in sex congruently as opposed to incongruently classified men, and significantly lower TIV in sex congruently as opposed to incongruently classified women (Fig. 3d and Table 2). Altogether, across both hold-out samples, this model tended to classify subjects with higher TIV as male and those with lower TIV as female, clearly indicating a brain size bias inherent in this model.

### Reducing TIV bias by confound removal

Featurewise control for TIV in the AM+ cr model resulted in decreased classification accuracies both for the AM (61.80%) and the ATM (72.98%; further details in Fig. 2, Table 1 and Table S2) hold-out samples. In comparison to the AM model with no TIV control (Fig. 3a) prediction probability displayed a much larger overlap between women and men (Fig. 3e, g). Further evaluation did not reveal any evidence for a TIV bias—i.e. neither did sex congruently classified men show higher TIV than incongruently classified men nor did sex congruently classified women show lower TIV than incongruently classified women in both the AM (Fig. 3f) and the ATM (Fig. 3h and Table 2) hold-out samples.

### Reducing bias by matching the training sample for TIV

The application of the two models built using TIV matched data with and without featurewise TIV control (ATM and ATM+cr model, respectively) to the AM hold-out sample resulted in similarly high classification accuracy (86.65% for ATM, 85.71% for ATM+cr model, details in Tables 1 and S2), performing between accuracies achieved by the AM and the AM+cr model. Thus, for the ATM models, additional featurewise TIV control did not result in decreased model performance. This is further reflected in similar prediction probability distributions (Fig. 3i, m), which were higher for men than for women. Likewise, the TIV of sex congruently and incongruently classified individuals did not differ significantly from each other both for women and for men (Fig. 3j, n and Table 2). Application of these models to the ATM hold-out sample (details in Tables 1 and S2), displayed better performance (92.55%) than for the AM hold-out sample. Furthermore, prediction probability distributions showed a comparable (Fig. 3k, o) but more pronounced pattern for the ATM hold-out sample. Again, when testing on the ATM hold-out sample, there was no difference between TIV of sex congruently and incongruently classified individuals both for the model without (Fig. 3l and Table 2) and with additional confound removal (Fig. 3p and Table 2).

Overall, the AM model achieved highest classification accuracy, but evaluation of the model output identified clear evidence for a TIV bias of the model. Reducing TIV-related variance by featurewise confound removal in the AM+cr model resulted in a less biased model, which also displayed a pronounced decrease in model performance, especially for the AM hold-out sample. Both models trained on the TIV balanced sample (ATM, ATM+cr model) did not show evidence of a TIV bias while still retaining high classification performance and appropriate calibration curves (Figs. S2 and S3), indicating that—at least for the present classification problem—training on a matched sample is more appropriate than featurewise confound removal. Thus, in the following, we will focus on comparing the performance of the biased AM model and the nonbiased ATM model on cisgender and transgender individuals in the application samples (sample A, sample B). Results for the AM+cr and ATM+cr models are provided in the Supplementary Results and Fig. S4.

### Biased performance of the AM model for cisgender and transgender individuals

**Figure 4:**
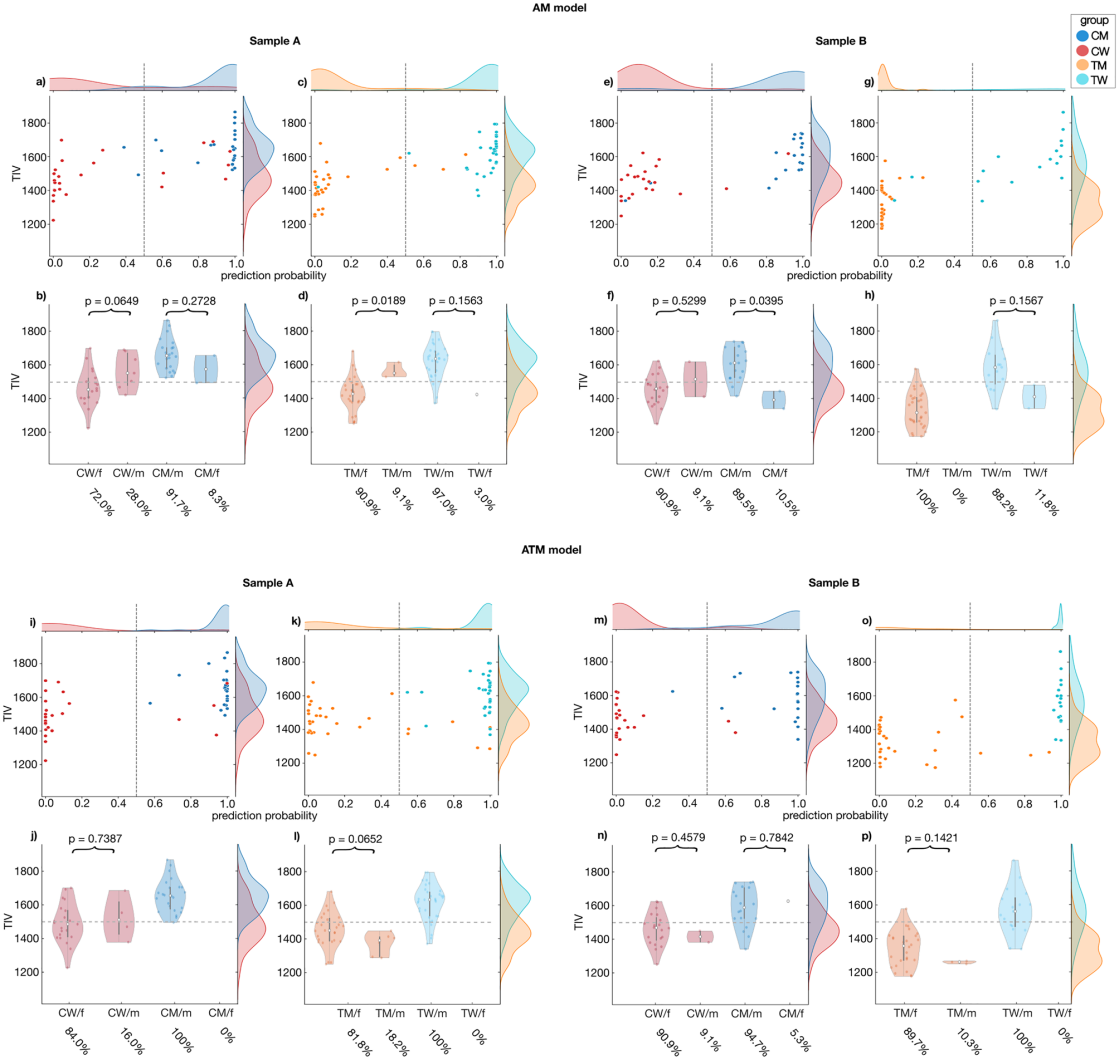
Association between prediction probability and TIV for the AM and ATM models in the two application samples. The upper row (**a**–**h**) shows the prediction probability (**a**,** c**,** e**,** g**) and TIV distribution (**b**, **d**, **f**, **h**) of sex congruently and incongruently classified CM, CW, TM and TW in the AM model in sample A and B. The bottom row (**i**–**p**) shows the prediction probability (**i**, **k**, **m**, **o**) and TIV distribution (**j**, **l**, **n**, **p**) of sex congruently and incongruently classified CM, CW, TM and TW in the ATM model in sample A and B. (CW/f: CW classified as female; CW/m: CW classified as male; CM/m: CM classified as male; CM/f: CM classified as female; TM/f: TM classified as female; TM/m: TM classified as male; TW/m: TW classified as male; TW/f: TW classified as female).

The application of the TIV-biased AM model resulted in an overall high performance of 88.70% for sample A, with an accuracy of 81.63% for cisgender and 93.43% for transgender individuals (detailed measures in Tables 1 and S3). Likewise, for sample B, the model achieved high overall accuracy of 93.10% (Tables 1 and S3) with an accuracy of 90.24% for cisgender individuals and 95.65% for transgender individuals. Matching the high accuracies, the prediction probability showed a sex congruent pattern with higher prediction probabilities for CM and TW (assigned male at birth) than for CW and TM (assigned female at birth) in both sample A (Fig. 4a, c) and sample B (Fig. 4e, g). A comparison of probability distributions of cis- and transgender individuals with the same sex revealed a trend for higher prediction probability for CW than for TM in sample A (*t* = 1.98, *p* = 0.0527, Cohen´s d = 0.53), which was significant in sample B (*t *= 3.58, *p* < 0.001, Cohen´s d = 1.01), matching the TIV-distributions showing higher TIV for CW than TM (Fig. S1).

The comparison of prediction probabilities for CM versus TW was not significant in both samples (Sample A: *t* = − 0.55, *p* = 0.5820, Cohen´s d = − 0.15; Sample B: *t *= 1.07, *p* = 0.2922, Cohen´s d = 0.36), while the effect size indicated a trend of lower prediction probability for TW than CM. While TIV-distributions for sex congruently and incongruently classified individuals did not differ significantly (Table 3), sex congruently classified CW and TM had a lower TIV than those classified in a sex incongruent manner. Sex congruently classified CM and TW had a higher TIV than those classified sex incongruently (Fig. 4b, d, f, h), indicating a similar bias of this model for both cisgender and transgender individuals.

**Table 3:** Wilcoxon rank sum tests of the application samples.

a)	**TIV CW classified as female versus classified as male**	**TIV CM classified as male versus classified as female**
AM model	*T* = 203, z = − 1.8459, *p* = 0.0649, η^2^ = 0.1363	*T* = 286, z = 1.0967, *p* = 0.2728, η^2^ = 0.0501
AM+cr model	*T* = 249, z = 0.8776, *p* = 0.3802, η^2^ = 0.0308	*T* = 236, z = − 1.0457, *p* = 0.2957, η^2^ = 0.0456
ATM model	*T* = 268, z = − 0.3336, *p* = 0.7387, η^2^ = 0.0045	*no CM classified as female*
AM+cr model	*T* = 268, z = − 0.3336, *p* = 0.7387, η^2^ = 0.0045	*T* = 294, z = 0.8668, *p* = 0.3861, η^2^ = 0.0313
	TIV TM classified as female versus classified as male	TIV TW classified as male versus classified as female
AM model	*T* = 472, z = − 2.3483, *p* = 0.0189, η^2^ = 0.1671	*T* = 558, z = 1.4178, *p* = 0.1563, η^2^ = 0.0609
AM+cr model	*T* = 477, z = 2.7689, *p* = 0.0056, η^2^ = 0.2323	*T* = 442, z = 0.6931, *p* = 0.4882, η^2^ = 0.0146
ATM model	*T* = 499, z = 1.8437, *p* = 0.0652, η^2^ = 0.1030	*no TW classified as female*
AM+cr model	*T* = 506, z = 1.4812, *p* = 0.1386, η^2^ = 0.0665	*T* = 532, z = 0.3395, *p* = 0.7342, η^2^ = 0.0035
b)	TIV CW classified as female versus classified as male	TIV CM classified as male versus classified as female
AM model	*T* = 224, z = − 0.6281, *p* = 0.5299, η^2^ = 0.0179	*T* = 186, z = 2.0591, *p* = 0.0395, η^2^ = 0.2231
AM+cr model	*T* = 199, z = 1.8328, *p* = 0.0668, η^2^ = 0.1527	*T* = 159, z = − 1.3948, *p* = 0.1631, η^2^ = 0.1024
ATM model	*T* = 237, z = 0.7424, *p* = 0.4579, η^2^ = 0.0250	*T* = 178, z = − 0.2739, *p* = 0.7842, η^2^ = 0.0039
AM+cr model	*T* = 237, z = 0.7424, *p* = 0.4579, η^2^ = 0.0250	*T* = 138, z = − 1.1500, *p* = 0.2501, η^2^ = 0.0696
	TIV TM classified as female versus classified as male	TIV TW classified as male versus classified as female
AM model	*no TM classified as male*	*T* = 145, z = 1.4162, *p* = 0.1567, η^2^ = 0.1180
AM+cr model	*T* = 289, z = 2.7714, *p* = 0.0056, η^2^ = 0.2648	*T* = 115, z = − 0.1698, *p* = 0.8651, η^2^ = 0.0017
ATM model	*T* = 411, z = 1.4680, *p* = 0.1421, η^2^ = 0.0743	*no TW classified as female*
AM+cr model	*T* = 411, z = 1.4680, *p* = 0.1421, η^2^ = 0.0743	*no TW classified as female*

Comparison of individuals classified as female versus male (Wilcoxon rank sum tests) for application sample A (a) and sample (b).

### Nonbiased ATM model: similar performances for cisgender and transgender individuals

The application of the ATM model to sample A displayed a high overall sex classification accuracy of 91.30% (91.84% for cisgender and 90.01% for transgender individuals). This model also performed accurately on sample B with an overall accuracy of 93.10% (92.68% for cisgender and 93.48% for transgender individuals, details in Table 1 and S3). In both samples, the ATM model yielded sex congruent prediction probabilities for all four groups (Fig. 4i, k, m, o). As opposed to the biased model, here, TM showed a trend of higher prediction probability than CW in Sample B (CW vs TM: *t* = − 1.27, *p* = 0.2093, Cohen´s d = − 0.36; Sample A: *t* = 0–0.47, *p* = 0.6425, Cohen´s d = − 0.12;). This gender congruent trend was not observed for TW (CM vs. TW: Sample A: t = 0.31, *p* = 0.7577, Cohen´s d = 0.08; Sample B: *t *= − 2.02, *p* = 0.0510, Cohen´s d = − 0.68). The comparison of TIV distributions between sex congruently and incongruently classified individuals (Fig. 4 j, l, n, p) did not reveal any significant differences (Table 3), neither for cisgender nor for transgender individuals, thus displaying no evidence for a TIV bias of this model.

## Discussion

In this work, we systematically compared two confound removal approaches, featurewise confound removal and sample stratification, with the aim to train accurate sex classification models without a TIV bias. In order to directly compare our findings to those of a previous study, we implemented a ML pipeline that has demonstrated high levels of sex classification accuracy^16^. This pipeline consisted of principal component analysis (PCA) for dimensionality reduction, followed by an SVM model with rbf kernel for learning, but did not report any consideration of the confounding effects of TIV.

Consistent with previous results, the baseline AM model which does not consider confounding effects of TIV achieved near-perfect classification accuracy on the AM hold-out sample by accurately classifying men with high TIV as male and women with low TIV as female^11,12,16,17^, but relied on TIV as a proxy for sex, indicating a pronounced TIV bias (Fig. 3b). The TIV bias was even more pronounced when the model was applied on the ATM hold-out sample presumably as the AM model was more likely to make mistakes for men with relatively lower TIV and women with relatively higher TIV. The pronounced TIV bias observed here is especially interesting, since the GMV data had already been scaled for TIV during preprocessing. Thus, our results align with previous claims that while the absolute amount of tissue is corrected for individual TIV, such scaling does not fully remove TIV-related variance (^32^, http://www.neuro.uni-jena.de/cat12/CAT12-Manual.pdf).

For the AM+cr model, where a featurewise removal of TIV was performed on the AM data, the misclassifications of both women and men were not systematically related to TIV differences, indicating that this model was not biased by TIV. This suggests that the AM+cr model based its classifications on different information than the AM model did. Our results match the findings of previous studies^20,30,33,34^, reporting a decrease in accuracy for sex classification models controlling for TIV in contrast to TIV-biased models. This decrease is likely related to the removal of TIV-related variance during featurewise confound removal, which might have decreased the overall amount of information available for the AM+cr model in contrast to the AM model^20,30,33,34^. This observation is in line with the results of a previous study suggesting that TIV alone contains enough information to classify sex at a similar level of accuracy as TIV-uncorrected GMV^34^. Considering that features in the AM sample can be assumed to contain more TIV-related variance than the ATM sample presumably explains why the drop in accuracy between the AM and the ATM+cr is less pronounced for the ATM hold-out sample than for the AM sample. Altogether, featurewise confound removal reduced TIV bias at the cost of classification accuracy. While a lack of bias in a model is desirable, so is high accuracy, suggesting that featurewise confound removal might not be the ideal approach to reduce TIV bias in structural sex classification.

In contrast to the models trained on the AM sample, both ATM trained models resulted in high and unbiased model performance for the AM as well as the ATM hold-out samples. The slightly higher accuracy for the ATM hold-out sample is likely due to the ATM hold-out sample better matching the characteristics of the ATM training sample, in particular with respect to TIV distribution, which is highly related to the target variable sex^30^. The better performance of the ATM and ATM+cr model on the ATM hold-out samples also supports the relevance of stratifying training and hold-out samples with respect to relevant variables that may interact with the target^35,36^.

The comparison of TIV of sex congruently and incongruently classified women and men did not indicate a TIV bias, which is in line with a study proposing beforehand matching to be a more efficient approach than feature-wise confound removal in the statistical analysis^9^. However, another study argued against the matching of data, arguing that matching for specific characteristics creates a sample that is not representative of the whole population^20^. While we agree that the ATM sample does not strictly represent the TIV distribution of the population by rather comprising men with relatively low and women with relatively high TIV, the ensuing models achieved high classification accuracies, even when applied to the AM hold-out sample which reflects the natural TIV distribution. This indicates that the models themselves are not biased by training sample characteristics, especially the restricted TIV range. In fact, the models appear to correctly capture sex differences in a generalizable manner as exemplified by their performance on the two hold-out samples. However, we would like to emphasize that both confound removal approaches employed in the present study rely on different statistical operations which are anticipated to result in different outcomes and model performances^8^. Thus, high model performance of one approach does not imply the other one to behave in a similar manner. For this reason, testing which approach is most suited for an individual ML-problem is crucial. The present results demonstrated that matching women and men for TIV in the training sample provides an appropriate approach for creating unbiased and accurate sex classification models.

In contrast to previous studies^16,17^, we observed similarly high classification accuracies for cis- and transgender individuals regardless of whether the models were debiased or not. This discrepancy may partly be explained by the fact that TIV of the transgender individuals in the present samples matched TIV of cisgender subjects of the same sex rather than aligning with gender identity (Fig. S1). Thus, even a biased classifier could accurately classify transgender individuals. However, in samples where the TIV values for transgender individuals indeed fall in-between those of cisgender men and women, as reported previously^25^ TIV-biased models would misclassify transgender individuals in accordance with their gender identity, which could explain prior findings^16^. Future studies should apply TIV-debiased models to additional datasets to help disentangle the complex interaction of sex, gender and the brain. It would be particularly interesting to apply our debiased models, which are available to other researchers (https://github.com/juaml/sex_prediction_vbm) to those datasets for which a reduction of sex classification accuracy for transgender participants has previously been reported^16,29^. Another explanation for the discrepancy between present and previous results^16,29^, might be that our classifiers learnt fundamentally different models, e.g. employing different feature weights than those in previous studies, which in turn might be caused by differences in characteristics of the training samples and in turn different parameters learnt during model optimization. Beside the differences due to different training samples, other factors affecting ML models and respective results might relate to differences in age-distribution. Here, we not only balanced for sex but also employed an exact matching of men and women with regards to age which might have reduced variance in comparison to the training-samples of other studies^16,29^ leading to differences in the fundamental model and results. In addition to age in the training sample, the age distribution of the application sample could also play a role, due to age-related GMV decline. Thus, older TW could be misclassified due to age-related GMV changes.

The present models were trained on a diverse collection of samples, ensuring a heterogeneity in several variables, such as age, scanning characteristics, and nationality. Likewise, as application samples we used two completely independent datasets comprising TW and TM. To our knowledge, previous studies have focused on test samples only comprising TW when applying a sex classifier trained on structural data of cisgender individuals to transgender individuals^16,29^, limiting conclusions to TW rather than transgender individuals in general. Notably, one study employing data of both TW and TM did not report significantly lower classification accuracy for transgender data^17^, which is in line with the present results. While we did not observe decreased sex classification accuracy for transgender individuals, this cannot be taken as a proof of absence of such structural brain differences, which might be revealed by the investigation of different sets of brain features or different analysis approaches.

Future studies can benefit by incorporating confound control approaches within interpretable ML pipelines that can provide insight into how many and which brain regions are most relevant for sex differences. Those insights can shed further light on which features are more common in men, women or both, thereby carrying implications for hypotheses as the mosaic of the human brain^37^, which exceeds the scope of the current study design. Methodologically sound studies, including both sex and gender aspects, are needed to improve our understanding of sex and gender-related differences in behavior and prevalence rates of mental disorders to advance development of sex-specific treatments^38,39^. Viewing patients through the lens of sex and gender is an essential step towards personalized care and individualized medicine^6,40^. Therefore, to achieve the ultimate goal of neuroimaging-based precision medicine, the present study takes a first step towards exploring appropriate confound removal in ML-based sex classification^41^. Although each ML analysis must consider confounds specific to the research question at hand, TIV is an important confound to consider in neuroimaging data in general, as also shown by others^9,18,33,34,42^. In addition to its application in sex classification analyses, as demonstrated here, appropriate confound control should also be considered for other ML applications. We, therefore, recommend that researchers should investigate which confound removal method is appropriate for their ML analysis.

## Conclusion

Our findings demonstrate that stratification via TIV-matching effectively eliminates TIV bias while achieving high levels of classification accuracy in a sex classification analysis using structural brain imaging features. Contrary to previous results^16^, our sex classification model demonstrated comparable levels of classification accuracy for both cisgender and transgender individuals. Our study emphasizes the importance of removing TIV bias appropriately in sex classification tasks to prevent incorrect interpretations. In general, confounding is a common issue in many ML-based modeling tasks, albeit with varying confounds and levels of confounding effects. Therefore, future studies utilizing ML approaches on brain imaging data should diligently examine for biases and implement appropriate confound control measures.

## Materials and methods

### Data

#### Data pool for model training and evaluation

To ensure a heterogeneous sample for training the classifiers, we combined data from 10 large cohorts into one data pool of structural magnetic resonance imaging (MRI) images from subjects differing in nationality, imaging parameters and age range. Supplementary Table S4 gives further details on the composition of the data pool, and details of the MRI data acquisition parameters can be found in the Supplementary Material. We only included subjects aged between 18 and 65 years with no indication of any psychiatric disorder, resulting in a total N of 5557 subjects. It is important to note, that the majority of large datasets, which have been employed for sex classification studies so far, likely report sex based on “presented sex”, i.e. the name and outer appearance of participants or on self-reported sex without explicitly collecting information on gender identity. We assume that among subjects not describing themselves as transgender, self-reported gender identity is equivalent to sex assigned at birth, while acknowledging that this match may neither be perfect nor binary.

Sixteen subjects whose TIV values differed more than three standard deviations from the mean TIV of the data pool were excluded as outliers. Then, two non-overlapping samples were extracted from the data pool. In the first sample (AM), women and men were matched for age to control for age-related GMV decline^43–46^. In the second sample (ATM), women and men were additionally matched for TIV. Possible differences between samples and sites in scanning acquisition were controlled for by including similar numbers of subjects from the different samples in the AM and ATM-sample respectively. Both the AM and ATM sample comprised 276 subjects from 1000 Brains, 146 subjects from Cam-CAN, 168 subjects from CoRR, 50 subjects from DLBS, 94 subjects from eNKI, 192 subjects from GOBS, 396 subjects from HCP, 96 subjects from IXI, 76 subjects from OASIS3, and 120 subjects from PNC. Each sample was split into a training (80%) and a hold-out sample (20%).

##### Age-matched (AM) sample

For the AM sample (*N* = 1614, 807 women), women and men were matched for age within each site (including multiple sites within one sample) by including a male counterpart from the same site whose age differed by no more than one year for each female subject. The age range in this sample was 18–65 years (*M* = 37.96*, SD* = 15.28). Further detailed information can be found in Table S1, and a plot of the TIV distribution of women and men is displayed in Fig. S1. There was no significant difference in age between women and men (*t* = 0.01, *p* = 0.99); however, the sexes differed significantly with respect to TIV (*t* = − 61.06, *p* < 0.001). Splitting the sample into training (80%) and hold-out samples (20%) resulted in 1292 subjects (646 women) for training and 322 subjects (161 women) for testing. The training and hold-out samples did not differ with respect to age (*t* = 0.98, *p* = 0.33) or TIV (*t* = − 0.11, *p* = 0.91). The age difference between sexes remained nonsignificant within both the training (*t* = − 0.00, *p* = 0.99) and the hold-out sample (*t* = 0.03, *p* = 0.97), whereas the TIV difference was significant for both samples (training: *t* = − 54.79, *p* < 0.001, hold-out: *t* = − 26.90, *p* < 0.001).

#### Age-TIV-matched (ATM) sample

For the ATM sample (*N* = 1614, 807 women), women and men were matched for age and TIV within each site. For each female subject, a male counterpart was included whose age differed by no more than one year and whose TIV differed by no more than 3%. The age range in this sample comprised 18–65 years (*M* = 38.15*, SD* = 15.35). More detailed information is displayed in Table S1, and the distribution of TIV for women and men in this sample is shown in Fig. S1. In this sample, women and men did not differ significantly in age (*t* = 0.01, *p* = 0.99), or in TIV (*t* = − 1.25, *p* = 0.21). The ATM sample was also divided into 80% for training and 20% hold-out for testing, again resulting in 1292 subjects (646 women) for training and 322 subjects (161 women) for testing. The training and hold-out samples did not differ with respect to age (*t* = 0.02, *p* = 0.98) or TIV (*t* = − 0.53, *p* = 0.60). Additionally, there was no significant difference between women and men in age or TIV in the training (age: *t* = 0.01, *p* = 0.99; TIV: *t* = − 0.99, *p* = 0.32) or hold-out sample (age: *t* = − 0.01, *p* = 0.99; TIV: *t* = − 0.83, *p* = 0.41).

#### Application samples

The first application sample (Sample A) was acquired in Aachen (Germany). This data set consisted of 115 individuals (24 CM, 25 CW, 33 TM, 33 TW). All cisgender participants were recruited via a public announcement around Aachen, whereas TM and TW were recruited in self-help groups and at the Department of Gynaecological Endocrinology and Reproductive Medicine of the RWTH Aachen University Hospital, Germany. All cisgender and transgender subjects in this sample reported no presence of neurological disorders, other medical conditions affecting the brain metabolism or first-degree relatives with a history of mental disorders. The Ethics Committee of the Medical Faculty of the RWTH Aachen University approved the study (EK 088/09,^23^). At the time of MRI measurement, 15 TM and 16 TW each were receiving hormone treatment. The age of the participants ranged from 18 to 61 years (*M* = 30.38, *SD* = 11.03). More detailed demographic information can be found in Table S1 and Fig. S1.

The second application sample (Sample B) consisted of an open-source dataset acquired in Barcelona, available via (https://data.mendeley.com/datasets/hjmfrv6vmg/2,^47–49^). The data set contained 87 subjects (19 CM, 22 CW, 29 TM, 17 TW) with an age range of 17 to 39 years (*M* = 22.23, *SD* = 4.97). More detailed information related to age and TIV in all four groups can be found in Table S1 and Fig. S1, though no information were available regarding the status of potential hormone treatment.

Model applications were evaluated on both application samples separately to further understand the model behavior on samples with differing characteristics (Table S1).

The data usage of the second application sample as well as the data for the AM and ATM-sample was approved by the Ethics Committee of the Medical Faculty of the Heinrich-Heine University Düsseldorf (2018-317, 4039, 4096, 5193). All subjects were participants in research projects approved by a local Institutional Review Board and provided written informed consent and all experiments were performed in accordance with relevant guidelines and regulations.

### Preprocessing of structural data

Structural T1-weighted MR images of all datasets were preprocessed using the Computational Anatomy Toolbox (CAT12.5 r1363, http://www.neuro.uni-jena.de/cat12/) in SPM (r6685) running under Matlab 9.0. After initial denoising (spatial-adaptive Non-Local Means), the pipeline included spatial registration, bias-correction, skull-striping and segmentation by an adaptive maximum a posteriori approach^50^ with using a partial volume model^51^. Subsequently, an optimized version of the Geodesic Shooting Algorithm^52^ was applied for normalization to MNI space and the resulting Jacobians were used for non-linear only modulation of grey matter segments, before final resampling to a 3 × 3 × 3 mm resolution via FSL. The non-linear only modulated images (m0wp1) were globally scaled for TIV internally with an approximation of TIV, i.e. every voxel was scaled by the relative linear transformation to the MNI152 template. Consequently, while TIV-related variance was likely not fully removed from the data, the GMV data included in the analyses were not fully TIV-naive.

### Predictive modelling

Whole-brain voxelwise GMV were used as features for training the classifiers, resulting in 77779 brain features (voxels) per subject. For each of the AM and the ATM training samples, classifiers were trained to predict sex with and without featurewise removal of TIV-related variance, resulting in the four different models: AM, AM+cr, ATM and AM+cr model (Fig. 1). For all four models, we employed a SVM classifier with rbf kernel^53^ using Julearn (https://juaml.github.io/julearn). Before training the classifier, PCA was performed to reduce the dimensionality of the data^16^. The maximum number of components (n = 1292, number of subjects in the training sample) was retained. Where applicable, for featurewise TIV control TIV-related variance was removed after dimensionality reduction by subtracting the fitted values of each feature in a cross-validation (CV)-consistent manner to avoid data leakage^20,30^. Stratified tenfold CV was performed to assess generalization performance. The two hyperparameters, C (1 − 1e^8^, log-uniform) and gamma (1e^-7^ − 1, log-uniform), were tuned via Bayesian Hyperparameter Optimization with 250 iterations within a fivefold CV inner loop following the analysis employed in a previous study^16^. The best performing combination of hyperparameters from the Bayesian Hyperparameter Optimization was used to train the final model on the full sample (details depicted in Supplementary Material).

The four final models were used to obtain predictions for the AM and ATM hold-out samples and both application samples (Fig. 1). Before application of the models to the hold-out samples, we ensured that the models were calibrated (https://scikit-learn.org/stable/modules/calibration.html#calibration) by assessing probabilities of classifying an individual into a respective class in relation to the actual labels of the individuals (Supplementary Figs. S2 and S3, Supplementary Results). These calibrations allow for checking whether the models gave accurate estimates of class probabilities and support probability predictions. To distinguish between the predicted and actual label of the sex a person identifies with, we refer to the terms “male” and “female” as predicted labels of an ML model whereas we refer to “men” and “women” as actual (true) label of an individual.

To further explore model behaviour, we compared the TIV-distributions of individuals classified in accordance with their sex and those who were not, by use of violin plots^54^ and by Wilcoxon rank sum tests. Due to the amount of comparisons conducted here, we chose a conservative significance level of α = 0.005 with effect sizes estimated accordingly^55^. To examine whether models were confounded by total GMV, we first tested whether GMV differed between the sexes in the two samples. In the AM sample, similarly to TIV, sexes exhibited significant differences in total GMV (two-sample t-test; t = − 31.21, *p* < 0.001). However, matching for TIV in the ATM sample also resulted in a non-significant difference in total GMV (t = 0.85, *p* = 0.40), indicating that matching on TIV was effective also for GMV. We then compared the GMV distributions of individuals classified correctly in accordance with their sex and those who were misclassified (Tables S5 and S6) with the same conservative significance level as for TIV-differences of α = 0.005. Further details can be found in the Supplementary Results and Tables S5 and S6. To assess potential differences between cis- and transgender individuals in prediction probabilities, we statistically compared probabilities of CM and TW as well as CW and TM. A power-analysis for these comparisons was conducted using G*Power to compute sample size required for effect sizes as found in previous work with a α–level of 0.05 and power-level of 0.8^29,56,57^.

### Supplementary Information

Supplementary Information.

## Data Availability

The data used in the study are available via open-source datasets, for which access information is provided in the supplementary information files together with the structural scanning parameter. Code is available on GitHub: https://github.com/juaml/sex_prediction_vbm.
